# A Novel Approach for the Shape Characterisation of Non-Melanoma Skin Lesions Using Elliptic Fourier Analyses and Clinical Images

**DOI:** 10.3390/jcm11154392

**Published:** 2022-07-28

**Authors:** Lloyd A. Courtenay, Innes Barbero-García, Julia Aramendi, Diego González-Aguilera, Manuel Rodríguez-Martín, Pablo Rodríguez-Gonzalvez, Javier Cañueto, Concepción Román-Curto

**Affiliations:** 1 Department of Cartographic and Terrain Engineering, Higher Polytechnic School of Ávila, University of Salamanca, Hornos Caleros 50, 05003 Ávila, Spain; ines.barbero@usal.es (I.B.-G.); juliaram@usal.es (J.A.); daguilera@usal.es (D.G.-A.); 2 Deptartment of Geology, Facultad de Ciencia y Tecnología, Universidad del País Vasco-Euskal Herriko Unibertsitatea (UPV/EHU), Barrio Sarriena s/n, 48940 Leioa, Spain; 3 Department of Mechanical Engineering, Universidad de Salamanca, 49029 Zamora, Spain; ingmanuel@usal.es; 4 Department of Mining Technology, Topography and Structures, University of León, 24401 Ponferrada, Spain; p.rodriguez@unileon.es; 5 Department of Dermatology, University Hospital of Spain, Paseo de San Vicente 58-182, 37007 Salamanca, Spain; javier.canueto@gmail.com (J.C.); croman@saludcastillayleon.es (C.R.-C.); 6 Instituto de Investigación Biomédica de Salamanca (IBSAL), Paseo de San Vicente 58-182, 37007 Salamanca, Spain; 7 Instituto de Biología Molecular y Celular del Cáncer (IBMCC)/Centro de Investigación del Cáncer (Lab 7), Campus Miguel de Unamuno s/n, 37007 Salamanca, Spain

**Keywords:** Non-Melanoma Skin Cancer, Elliptic Fourier Analysis, shape analysis, skin lesion asymmetry, clinical images, computer vision

## Abstract

The early detection of Non-Melanoma Skin Cancer (NMSC) is crucial to achieve the best treatment outcomes. Shape is considered one of the main parameters taken for the detection of some types of skin cancer such as melanoma. For NMSC, the importance of shape as a visual detection parameter is not well-studied. A dataset of 993 standard camera images containing different types of NMSC and benign skin lesions was analysed. For each image, the lesion boundaries were extracted. After an alignment and scaling, Elliptic Fourier Analysis (EFA) coefficients were calculated for the boundary of each lesion. The asymmetry of lesions was also calculated. Then, multivariate statistics were employed for dimensionality reduction and finally computational learning classification was employed to evaluate the separability of the classes. The separation between malignant and benign samples was successful in most cases. The best-performing approach was the combination of EFA coefficients and asymmetry. The combination of EFA and asymmetry resulted in a balanced accuracy of 0.786 and an Area Under Curve of 0.735. The combination of EFA and asymmetry for lesion classification resulted in notable success rates when distinguishing between benign and malignant lesions. In light of these results, skin lesions’ shape should be integrated as a fundamental part of future detection techniques in clinical screening.

## 1. Introduction

Non-melanoma skin cancer (NMSC) is one of the most common malignancies, with an especially high incidence rate among elderly and white-skinned populations. NMSC includes different pathologies, with Basal Cell Carcinoma (BCC) and Squamous Cell Carcinoma (SCC) being the most common. The early detection and diagnosis of NMSC reduces the risk of bad prognoses, as well as the costs these pathologies entail on health systems due to their high incidence [1,2].

The first step in skin cancer diagnosis, including melanoma and NMSC, is a visual examination. For the detection of melanoma, the ABCDE mnemonic is a widely used tool [3,4], which considers the following variables: Asymmetry, Border irregularity, Colour uniformity, Diameter, and Evolution. In ABCDE, shape can be considered a combination of asymmetry and border irregularity. Other identification methodologies also include parameters that can be considered an important component of overall morphology, such as border irregularities, as presented by MacKie [5].

For NMSC, the dermoscopic features of its lesions are well-studied [6]. Nevertheless, studies taking shape into consideration are very limited, while this parameter is mostly evaluated in combination with others, such as lesion colour and texture [7].

While asymmetry is considered an important parameter for the visual detection of skin cancer, there is a lack of empirical data that relates the shape of the lesion to the probability of it being malignant. From this perspective, an objective characterisation and definition of lesions’ shape may not only be useful for visual examination but could also aid the development of more precise and non-invasive methodologies. This variable can additionally be integrated into more developed methodologies using images and Artificial Intelligence, a field of research that has been of growing importance over the last few years [8,9].

The study of morphology is of growing interest in several fields of science [10], fueled primarily by the integration and improvement of advanced computer vision techniques towards the manipulation of different types of data. Many methods exist for the study of morphology, varying mostly by the means in which the data are defined and extracted. One common approach to the study of morphology is that of Geometric Morphometrics (GMM) [11,12,13]. GMM is a growing protagonist in fields related to biology and evolution [10], with other interesting applications in forensic sciences [14,15] and the study of microscopic elements on bones [16,17,18]. Nevertheless, GMM analyses are often hindered by the definition of landmark data; landmarks are precise homologous loci, of biological or geometric significance, that must always be identifiable across the sample [12,19].

In response to this, analysts began developing a different yet closely linked approach, making use of Fourier descriptors as a function of shape [20,21,22]. The principle of Fourier Analyses (FA) is to describe shape as a series of periodic functions along the curvature of an outline [21]. From this perspective, FA overcomes the limitations presented by GMM approaches, providing a means of analysing forms without a strict definition of homologous landmarks [11,13]. This type of methodology has been employed in a wide array of applications, ranging from the study of leaf shapes in biology [23], anthropological applications [24], or even the analysis of object design over time [25,26].

In this study, we present a novel approach to analysing skin lesions’ shape, employing FA to investigate the shape of different skin lesion outlines. Thus, the aim of this research is to highlight the possible differences among NMSCs and benign skin lesions, proposing shape as a useful parameter for skin lesion classification. From this perspective, the data presented may provide an empirical approximation to the characterisation of skin lesions’ shape.

## 2. Materials and Methods

### 2.1. Image Dataset

**Figure 1:**
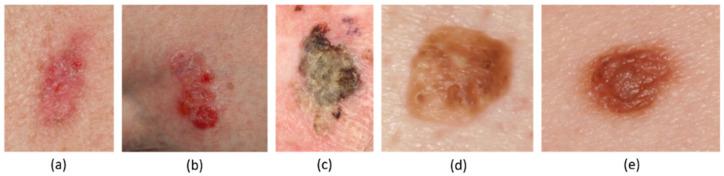
Example images for the different skin lesions, including BCC (**a**), IEC (**b**), SCC (**c**), and BEN: Seborrhoeic Keratosis (**d**) and Melanocytic Nevus (**e**).

The images used for the analysis were obtained from the Dermofit Dataset [7] (Figure 1), provided by the University of Edinburgh. This dataset has proven to be useful for the training of neural networks for skin lesion classification [27,28] and the segmentation of images via generative adversarial networks [29]. The scale of each image is unknown, and they were taken using a standard camera, thereby covering the visible area of the electromagnetic spectrum. No dermoscopes were employed for the collection of data. The Dermofit dataset additionally contains a mask delimiting each lesion area.

The original dataset consists of 10 classes, covering different cutaneous lesions. For the present study, the number of classes included was reduced, giving preference to well-defined NMSC lesions, and joining benign pathologies into one class, as a distinction between them was not considered clinically relevant. Under this premise, this study analyses a total of 4 different skin lesion types: Basal Cell Carcinoma (BCC, *n* = 239; Figure 1a), Intraepithelial Carcinoma (IEC, *n* = 78; Figure 1b), Squamous Cell Carcinoma (SCC, *n* = 88; Figure 1c), and a collection of benign lesions (BEN, *n* = 588), joining Seborrhoeic Keratosis (Figure 1d) and Melanocytic Nevus (Figure 1e).

### 2.2. Definition of Lesion Boundaries

**Figure 2:**
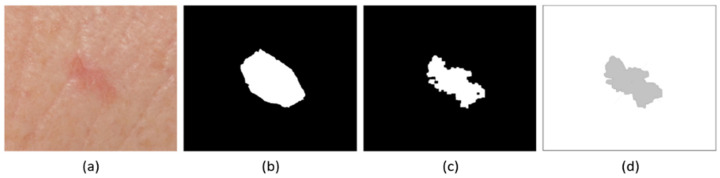
Example of original image (**a**), lesion mask provided as part of Dermofit dataset (**b**), recalculated lesion mask using k-means clustering (**c**), and obtained lesion boundary (**d**).

To define the borders of the lesions, a combined approach was followed. First, the original image segmentation provided by the Dermofit dataset was considered. This segmentation was manually established by the medical experts who curated the dataset. Nevertheless, in some cases, these classifications were found to have important differences regarding visual segmentation (the visual appearance of lesions; Figure 2a), resulting in a simplification of the boundaries (Figure 2b). The expert definition of boundaries is considered the optimal segmentation from a medical point of view; nevertheless, pixel level analyses to fit these boundaries to the point of highest spectral change can yield a higher level of detail and precision, thus providing a more empirical definition of the visual shape of the lesion. From this perspective, the automated refinement of segmentations using computer vision-based techniques allow for a more reproducible segmentation of the image, while the criteria given by the dermatologist remains crucial.

To obtain pixel-level segmentation, the present approach modified the manual technique by including an automatic computer vision technique. For this, each image was segmented using a k-means clustering algorithm [30] with 4 classes, defining areas mostly inside the pre-established boundary as a lesion, and thus refining the manual segmentation, based on their characteristics in the visual spectrum. Then, this was followed by a morphological closing algorithm [31], removing isolated areas and thus cleaning the segmented image to provide a single outline. This technique facilitated a better definition of lesion borders, especially in images where manual segmentation was observed to not fit well around the visual edges of the lesion (Figure 2b,c).

The obtained lesion boundary for each image was defined by 300 points, which were considered enough for a detailed representation of shape (Figure 2d).

Segmentation processes were performed using the Python programming language (v.3.7.6) and the OpenCV library.

### 2.3. Elliptic Fourier Analyses

Once outlines had been extracted, geometries were aligned and centered using variance–covariance matrices and eigenvalues. This step ensures that further calculations are invariant of the outline location, rotation, and origin. Outlines were also scaled using geometry centroid sizes as a scale factor (measured in pixels) to ensure pixel size and camera distance were not conditioning factors for the description of morphology. Centroid size was calculated as the distance from the edge to the centre (centroid) of each lesion along multiple points along the outline. Size features could not be further considered in the analyses because the Dermofit dataset does not provide a scale bar for each photo, which also obstructs any type of analysis of the lesion’s *form* (shape + size; [32]). After normalisation procedures, outlines were analysed using an FA approach.

Fourier series are used to describe shapes by decomposing a periodic function into a sum of simpler trigonometric functions, such as sine and cosine values. These periodic functions can consider: (1) the distance of any point on the outline to the centroid [33], (2) the variation of the tangent angle for any point [33], or (3) a series of linearly transformed circular coordinates [34,35]. These approaches are known as Fourier Radius Variation, Fourier Tangent Angle, and Elliptic Fourier analyses, respectively. While each approach has its advantages and disadvantages, Elliptic Fourier Analyses (EFA) are more robust to irregularities along the outline [34,35] without the need for points to be equally spaced, thus enabling EFA to be easily fitted to any type of geometry. For this reason, EFA was selected as the most optimal approach for the present study.

Once calculated, each of these periodic functions can then be decomposed using Fourier series, resulting in a harmonic sum of trigonometric functions weighted with harmonic coefficients. Using EFA, Fourier coefficients are divided into 4 main groups, labelled *a* through *d*. Coefficients *a* and *b* can be simply defined as the trigonometric moments around *x* coordinate values, while coefficients *c* and *d* define the *y* coordinate projection from circular to linear space [21,34,35]. Depending on the number of harmonics (*n*) used to describe the Fourier series, a set of coefficients—*a_n_*, *b_n_*, *c_n_* and *d_n_*—can then be subjected to multivariate statistical analyses to empirically define each outline.

### 2.4. Multivariate Statistics

For the present study, the first 19 harmonics of the elliptical Fourier series were used as descriptors of skin lesions’ outlines. The optimal number of harmonics was calculated by estimating the cumulative power for each harmonic, with 19 harmonics representing up to 98.3% of the cumulative power. As is common practice in EFA, coefficients *a*_1_, *b*_1_, and *c*_1_ were then used to normalise data [21], eliminating any remaining influence that size or rotation may have on subsequent analyses. This resulted in a final dataset of 73 morphological descriptors per individual.

Following this, dimensionality reduction was performed across coefficients through Principal Components Analyses (PCA). Principal Component (PC) Scores were then carefully assessed to evaluate the percentage of variance represented, selecting only those PC scores representing up to 95% of variance. Following PCA, analyses were carried out to assess the homogeneity of sample distributions using multiple Shapiro tests [36]. If samples were found to fit a Gaussian distribution, then subsequent analyses adopted a parametric approach, while non-Gaussian distributions were studied using robust statistical methods [8,37].

To assess statistical differences and similarities among groups, Multivariate Analyses of Variance (MANOVA) were performed. For normally distributed PC scores, the Hotelling–Lawley test statistic was used [38]. When normality was rejected, robust alternatives such as the Wilk’s Lambda test statistic were used [39].

Additional analyses considered the use of Mahalanobis distances. For this purpose, within-group covariance distributions were first calculated, and then compared with distances to members of other groups. Statistical assessments of distribution differences were performed using either univariate Analysis of Variance tests (ANOVA) or Kruskal–Wallis tests, for Gaussian and Non-Gaussian distributions, respectively [40].

Changes in outline shape were visualised with the aid of transformation grids and warpings, computed using Thin Plate Splines (TPS) [41]. TPS grids minimise the bending between shapes to express changes in the relative position of points along the outline as the deformation of a grid. Therefore, TPS were used to fit central shape configurations for each of the groups separately and to visually calculate deformations when compared with other samples. Final calculations of outline deformations were then performed with the help of an isoline contour function. Additionally, oscilloscopes were used to evaluate changes in x and y coordinates across outlines. A trapezoidal integration was then computed to approximate an estimation of the area of each function (α), thus evaluating the smoothness of oscilloscope curves. To provide a frame of reference, a perfect theoretical ellipsoid was computed to have an α = 0.0.

Considering recent criticism on the “blind” use of *p*-values in applied statistics, the present study evaluated the hypothetical results while excluding the *p* < 0.05 rule for defining “statistical significance”. In accordance with the most recent recommendations set forth by the American Statistician [42,43], *p*-values were evaluated by accompanying calculations of the probability of observations being a Type I statistical error, or the False Positive Risk (FPR) [44]. FPR values were calculated using the Sellke–Berger approach to define the likelihood ratio of the null hypothesis against the alternative hypothesis [45,46]. In general, prior probabilities of 0.5 were used for *p*-value calibrations, as suggested by Colquhoun [44,47]. Nevertheless, where possible, calibration confidence intervals were constructed using prior probabilities of 0.8 and 0.2 as well [8]. Throughout the study, FPR value calculations were only excluded for *p*-values over a 0.368 threshold, considering these values to be too high to accept the alternative hypothesis on any grounds [8]. Finally, a more robust *p*-value threshold of 0.003 was adopted as a threshold for more conclusive results, considering how this value is 3 standard deviations (3σ) from the mean, and associated with a 4.5% chance of being a Type I statistical error when using 0.5 prior probabilities [8].

All statistical applications were performed in the R (v.4.0.4) programming language. The R code to calculate EFA coefficients is available in the Supplementary Materials. Visualisations of EFA results were performed, in part, using the Momocs R library [48].

### 2.5. Asymmetry Calculations

To empirically quantify and analyse lesion asymmetry, an index was designed and implemented. For each lesion, the centroid was calculated and then used to transpose outlines so that the x or y axis aligned with 0. Once centered, the absolute values of the axis in question were calculated, removing the line of “symmetry” between each value (*x* or *y*) and the corresponding point on the opposite side of the outline (*x*′ or *y*′). The Euclidean distance, *d* (*x_i_*, *x*′*_i_*), was then calculated between each point, and used to derive a quantitative measurement of outline displacement. An asymmetry index (*a*(*x*) or *a*(*y*)) was then assigned to axis *x* and axis *y*, respectively, through the root mean square Euclidean distance across each outline (Equation (1));

(1) $$a\left(x\right)=\sqrt{\frac{1}{n}\overset{n}{\underset{i=1}{\sum }}d{\left(x_{i},\left|{x^{\prime }}_{i}\right|\right)}^{2}},\;a\left(y\right)=\sqrt{\frac{1}{n}\overset{n}{\underset{i=1}{\sum }}d{\left(y_{i},\left|{y^{\prime }}_{i}\right|\right)}^{2}},$$

The final asymmetry index for each skin lesion was calculated as the maximum index among the *x* and *y* axes.

Once asymmetry indices had been obtained for each sample, samples were tested for normality using Shapiro–Wilk tests, and then described using either traditional or robust statistical approaches [8,37,49]. For traditional descriptive statistics, sample mean and standard deviation were used to calculate central tendency and dispersion, respectively. For robust statistics, these were replaced by the median and the Square Root of the Biweight Midvariance (√BWMV). Similarly, 95% confidence intervals were also constructed using a [0.05, 0.95] interquartile range. Next, distributions of samples were analysed for statistical differences via ANOVA or Kruskal–Wallis tests. In addition to this, all aforementioned Fourier analyses were then repeated incorporating the asymmetry index into PCA, including the calculation of multivariate differences through MANOVA and Mahalanobis distances.

### 2.6. Machine Learning

To test the degree of separation amongst samples, classification tasks were performed using machine learning techniques. Therefore, a *k*-fold cross-validated (*k* = 10) Support Vector Machine (SVM) with a Radial Basis Function (RBF) was used [50]. SVMs are customizable and flexible models that use a *kernel-trick* to adjust for the existence or inexistence of parametric components, such as normality. Thus, this *kernel-trick* allows SVMs to construct non-linear decision boundaries. The SVM is additionally characterised using a soft maximised-margin as a decision boundary, thus avoiding overfitting of the data used for training.

**Figure 3:**
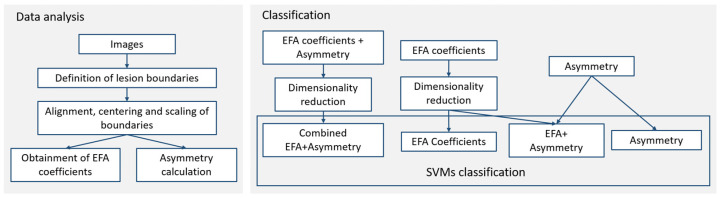
Methodological workflow.

SVMs were trained on 70% of data, separating the remaining 30% for testing and model evaluation. SVMs were mostly trained on raw PC scores, filtering only those PC scores representing up to 95% sample variance. For this purpose, the first experiment trained SVMs on PC scores obtained from EFA coefficients (Figure 3), while the second experiment trained SVMs on PC scores calculated when asymmetry indices were also included. Nevertheless, two additional experiments were also performed (Figure 3): one calculating the degree of univariate separability on asymmetry indices alone, and a final experiment appending the PC scores obtained from EFA coefficients with the asymmetry indices, enabling an assessment of the effect asymmetry has on classification results prior to a combined dimensionality reduction (Figure 3).

For the selection of each SVM’s optimal cost (*c*) and gamma (γ) hyperparameters, Bayesian Optimization Algorithms (BOAs) were employed [51,52,53]. BOA was initialised using a random optimization algorithm, thus defining the prior distribution for hyperparameter selection [53]. This was then followed by an Expected Improvement (EI) BOA algorithm for 50 iterations. While Gaussian Process Upper Confidence Bound (GPUCB) and Probability of Improvement (PI) selection functions were also experimented with, they did not provide notable differences from their EI counterpart [53,54].

SVMs were evaluated on test sets, taking into consideration the general balance and imbalance of different sample sizes within the dataset while choosing appropriate evaluation criterion. While the selection of lesions from the Dermofit dataset does not present an extreme imbalance between benign and malignant tumours (≈29:20), when comparing between individual samples, this imbalance increases greatly (≈97:13 in the worst of cases). From this perspective, the present study chose to use evaluation metrics less susceptible to changes in sample balance [55], namely, Accuracy, Precision, Recall, the F1 Score, and the Area Under the precision–recall Curve (AUC). Each of these metrics, except for AUC, were calculated using confusion matrices, measuring the ratio of correctly classified individuals (True Positive & True Negative), as well as miss-classified individuals (False Positive & False Negative). AUC curves were calculated on the probability of label association values.

Machine learning applications were performed in the R programming language (v.4.0.4), primarily using the “caret” library.

## 3. Experimental Results

### 3.1. Elliptic Fourier Analyses

**Figure 4:**
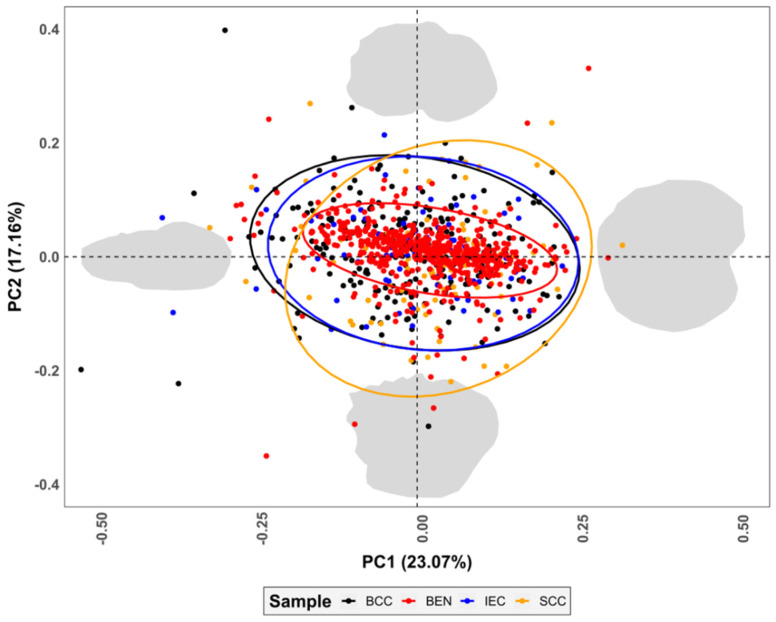
Principal Component Analysis (PCA) scatter plots with 95% confidence intervals presenting variance in skin lesions’ shape, as represented by Elliptic Fourier Analyses. Morphological variance calculated through Thin Plate Spline grid warpings are presented at the extremity of each PC score in grey. Shape space coordinate (0,0) is represented by circular lesions with no border irregularities. BCC = Basal Cell Carcinoma, BEN = Benign, IEC = Intraepithelial Carcinoma, SCC = Squamous Cell Carcinoma.

The analyses of the skin lesion morphologies revealed border irregularity to be a fundamental descriptive component of mainly malignant tumours. In general, PCA dimensionality reduction produces a high number of inhomogeneous PC scores (Shapiro *w* = 0.86, *p* = 1.1 × 10^−^^28^, FPR = 2.0 × 10^−^^24^%), with the first 15 PC scores representing ≈90% of variance and 21 PC scores reaching ≈95% cumulative variance. The PCA plots (Figure 4) reveal a strong concentration of benign lesions (red colour in Figure 4) in the centre of each dimension (median [*x*, *y*] shape space coordinates = [0.009, 0.0009]), represented by more circular lesions, while all three malignant samples present much higher variance across the shape space (√BWMV Benign = 0.094 and Malignant = 0.115).

**Figure 5:**
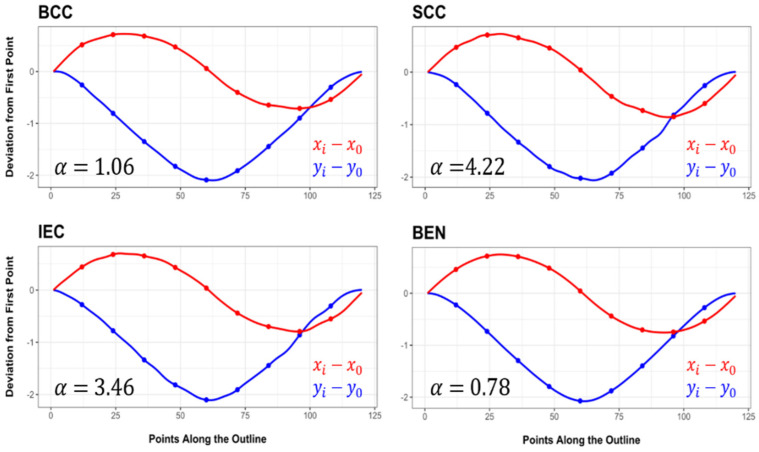
Oscilloscope curves reflecting variations along the outline of each of the samples according to elliptical Fourier descriptors. α values represent the results obtained from computing the area of each oscilloscope function. Perfect elliptical outlines would be presented by smooth sinusoidal curves with no irregular deviations (α = 0.0).

Upon analysing the projection and the morphological variations along the curvilinear abscissa (Figure 5), the oscilloscopes confirmed Benign samples to be the most elliptical lesions in nature (α = 0.78), with hardly any deviations from a theoretical ellipsoid (Figure 5). The SCC (α = 3.46) and IEC (α = 4.22) samples, on the other hand, appear to deviate the most along the outline, with frequent irregularities along the lesion borders. Interestingly, the BCC samples present a relatively smooth curve, where the malignant samples are of the greatest spherical nature (α = 1.06).

**Figure 6:**
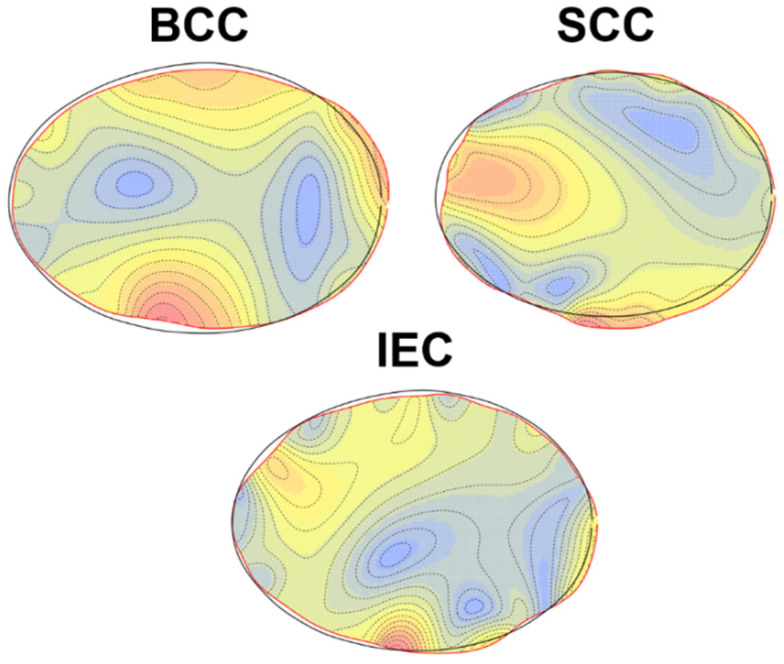
Deformation grid visualisations via isoline plots, projecting each of the central configurations for malignant samples onto the central shape of benign skin lesions. Red areas reflect areas of greater deformation from benign samples.

When analysing the differences between each of the malign lesions in comparison with the benign samples, the Thin Plate Splines (TPS) and isoline plots confirm these deformations (Figure 6), with samples such as SCC and IEC presenting distinct lateral constrictions. The BCC samples, on the other hand, are characterised by a more irregular-oval shape. Overall, the isoline heatmaps reveal all the malignant samples to present highly localised deformations, which would indicate shape variation to be a product of edge irregularities, as opposed to an overall change across the entire elliptical nature of the lesion. From this perspective, it could be assumed that lesion asymmetry is a powerful conditioning factor in diagnoses of malignant and benign lesions.

The multivariate quantification of the sample differences based on EFA shows that benign lesions frequently separate from all three types of malignant samples (MANOVA *p* = 0.002, FPR = 3.3 +/− [0.8, 11.9]%). When considering the Fourier coefficients alone, the separation between the Benign and IEC samples becomes a little less clear (Table 1), with a 5.7 +/− [1.5, 19.4]% chance of being a Type I statistical error when using MANOVA testing. Similarly, while the MANOVA results hint towards a possible separation among some of the malignant samples, the FPR calculations are too high to consider these observations conclusive, indicating that the malignant tumours are morphologically similar among themselves.

When considering the Mahalanobis distances (Table 1), the calculations reveal much larger differences between the sample distributions, especially when separating between the Benign and Malignant lesions as a whole (*p* = 2.5 × 10^−74^, FPR = 1.2 × 10^−69^ +/− [2.9 × 10^—^^70^, 4.6 × 10^−69^]%). In this case, none of the malignant lesions appear to be similar, while benign lesion multivariate distributions appear to be notably separate from each of the carcinoma samples.

### 3.2. Lesion Asymmetry

**Figure 7:**
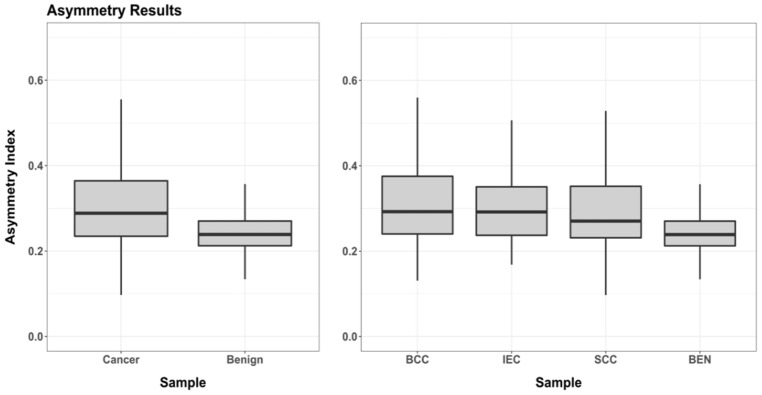
Boxplots presenting the maximum asymmetry index calculations for each of the samples.

Upon calculating the asymmetry indices for each of the samples, great differences emerged between the benign lesions and each of the carcinoma samples (Table 2, Figure 7). In most of the cases, the malignant lesions present much higher variability (Interquantile Range = 0.37, √BWMV = 0.093) as opposed to benign lesions (Interquantile Range = 0.19, √BWMV = 0.046). The differences between these samples are also of great importance (χ^2^ = 103.3, *p* = 2.2 × 10^−16^, FPR = 2.2 × 10^−^^12^ +/− [5.4 × 10^−13^, 8.6 × 10^−12^]%). When considering each malignant sample separately, boxplots indicate that BCC is the sample with the greatest degree of asymmetry (Figure 7); nevertheless, robust metrics (Table 2) reveal the IEC and BCC to have the same central index, with IEC presenting the largest robust interquartile range (BCC = 0.375, SCC = 0.400, IEC = 0.435).

**Figure 8:**
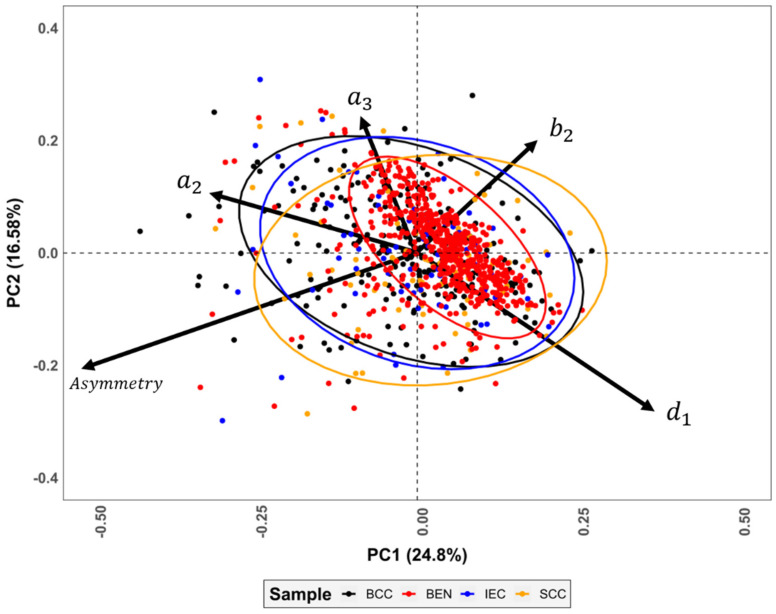
PCA biplot combining asymmetry indices with elliptical Fourier coefficients. For visual simplicity, only the first 5 most important variables were included in the biplot. Variables a, b, and d represent elliptic Fourier coefficients.

Integrating asymmetry indices into the multivariate statistical analyses produces similarly complex non-gaussian PCA distributions (*w* = 0.86, *p* = 1.1 × 10^−28^, FPR = 2.0 × 10^−24^%), with ≈90% of the cumulative sample variance appearing in the first 15 PC scores and ≈95% in the first 21. As can be seen in the sample biplots (Figure 8), the asymmetry index represents the variable of greatest importance in the description of the sample morphology, correlating strongly with both PC1 (24.8% variance, *p* = 2.1 × 10^−238^, FPR = 3.1 × 10^−233^%) and PC2 (16.6%, *p* = 6.5 × 10^−24^, FPR = 9.5 × 10^−20^%).

Through an in-depth analysis of the PCA scatter plots, it was observed that the asymmetry index produces a notable irregular dispersion among the malignant samples (skewness = −3.0, kurtosis = 12.0), pushing the benign lesions into a much more concentrated distribution (skewness = −3.9, kurtosis = 33.8), which is better described by the original elliptic Fourier coefficients.

As opposed to the calculations performed on Fourier coefficients alone, the inclusion of asymmetry presents a notable improvement in both the MANOVA and Mahalanobis results (Table 3), with all malignant lesions appearing clearly separable from benign lesions (MANOVA *p* = 0.001, FPR = 1.8 +/− [0.5, 7.0]%; Mahalanobis *p* = 3.6 × 10^−75^, FPR = 1.7 × 10^−70^ +/− [4.2 × 10^−71^, 6.7 × 10^−70^]%).

Thus, all the multivariate statistical results conclude asymmetry to be a considerable component for the identification of malignant lesions, with benign lesions being mostly characterised by their elliptical shape and greater overall symmetry.

### 3.3. Machine Learning

The SVMs were found to successfully learn from the morphological data on most accounts (Table 4), with the worst performing models being univariate SVMs trained solely on asymmetry indices. The evaluation metrics also concur with the multivariate statistical results, revealing the combination of EFA data with the asymmetry index to be the most efficient means of differentiating between malignant and benign tumours (Table 5). Interestingly, SVMs appear to identify benign lesions with much greater accuracy than malignant lesions. This is likely because not all malignant lesions present an irregular border, while a much greater percentage of benign lesions are found to be concentrated around the elliptical-symmetric portion of the shape space. Nevertheless, the true positive to true negative rates remain high, resulting in a fairly balanced AUC metric.

## 4. Discussion

A well-known feature for the characterisation of malignant and benign skin lesions is their shape. While most diagnostic criteria try to assess these variables as a function of border regularity and overall symmetry, few studies have tried to empirically quantify these morphological traits. The present study analyses the morphological differences amongst NMSC and benign lesions using Fourier descriptors and asymmetry calculations. To the authors’ knowledge, this is one of the first approximations to objectively defining these dermatological criteria using computer vision and multivariate statistical techniques.

In recent years, morphological tools such as GMM and EFA have proven highly efficient in the evaluation of medical data. From this perspective, interesting studies have employed landmark-based techniques for the diagnosis and evaluation of patients with several diseases and syndromes. These include, but are not exclusive to, Beta Thalassaemia [56], Glut1 Deficiency Syndrome [57], Fetal Alcohol Syndrome [58], Obstructive Sleep Apnea Syndrome [59], and (though not strictly using GMM) the study of Down Syndrome patients [60]. Fourier descriptors, on the other hand, have been used to a lesser extent, with applications in ovarian tumour analysis [61], as well as the study of optic nerve head morphology and glaucoma [62]. The present study contributes to these efforts, expanding dermatological analyses to include these types of tools as well.

While GMM has proven to be more popular in medical analyses over EFA, this is likely due to the large corpus of pre-existing research using GMM in the analysis of cranial morphology in physical anthropology [10]. From this perspective, the definition of landmarks for these types of analyses are already well-defined, while post-cranial soft-tissue research in medicine is notably lacking. Considering the difficulties that may exist in defining truly homologous landmarks on soft tissue, EFA presents the distinct advantage of being able to describe morphological data in elements where landmarks may not exist. Nevertheless, a fundamental component in any of these studies is the method though which these data are obtained.

A correct and objective definition of skin lesions’ boundaries is a complex task, with most advanced techniques involving methodologies such as spectroscopy or hyperspectral imaging [8,29,63,64]. The task is especially challenging given the nature of the images used for this study, covering only the visible area of the spectrum. Similarly, while benign samples are composed mostly of pigmented lesions, NMSC classes often contain un-pigmented lesions, whose delimitation is especially complex. For this reason, the present study worked with a dataset of visually delimited lesions, whose boundaries could later be refined using K-means algorithms. From this perspective, future research should address the use of morphological analyses on boundaries that have been extracted using automated methods. These could include techniques such as those provided when combining multispectral or hyperspectral imagery, alongside advanced computational learning techniques.

Employing image segmentation techniques on ultrasound images, a methodologically similar study by Martínez-Más and colleagues [61] was able to successfully characterize ovarian tumours, reaching up to ≈87% accuracy and an ≈0.87 Area under the Receiver Operator Characteristic Curve. While applied to a different medical case study, these authors present an additional account of how Fourier shape descriptors and machine learning algorithms can be considered useful tools in medical diagnostics.

The present study revealed notable statistical differences between benign and malignant skin samples, wherein most of the statistical tests appeared conclusive (FPR < 6%). In addition, machine learning algorithms were able to reach up to 78.6% accuracy (AUC = 0.735). If integrated into a practical tool, combining both the use of automated outline extraction using computer vision techniques and the additional analyses of these outlines via Fourier shape descriptors, this methodological workflow could prove to be a powerful tool, especially at the screening stage of skin cancer diagnoses.

Clearly, the present results reveal shape and asymmetry to be more of an indicator of malignancy than the type of malignancy. The results obtained within this study hint that all malignant cutaneous tumours are mainly characterised by morphological irregularities in comparison with asymmetry, while not much else can be obtained through the current methodology. Nevertheless, it is important to note that most diagnostic criteria in skin lesion research are based on a combination of variables [3,4,5] and no single variable alone. At present, the EFA approaches described herein have been limited to a description of pure shape, while the lack of a scale bar in the Dermofit dataset hinders the possibility of studying form (shape & size; [32]). Likewise, an important component of skin lesion diagnostics is found in colour [3,4,5], a variable that may be integrated into future analyses through more advanced computer vision techniques.

In conclusion, this study describes a new methodological approach to the characterisation of non-melanoma- as well as benign-type skin lesions. Through a combined use of computer vision techniques, elliptical Fourier analyses, and computational learning, a ≈79% separation has been achieved between malignant and benign lesions, supported by notable statistical results (*p* < 0.003). Similarly, asymmetry has been found to be a fundamental variable in the description of cutaneous carcinomas. Nevertheless, future investigation should be dedicated to the analysis of more efficient and accurate segmentation procedures, while searching for means to integrate morphological and electromagnetic information into a more robust and well-rounded diagnostic tool.

## Figures and Tables

**Table 1:** Multivariate Analysis of Variance (MANOVA) and Mahalanobis distance testing to assess the degree of statistical differences between sample outlines. BCC = Basal Cell Carcinoma, BEN = Benign, IEC = Intraepithelial Carcinoma, SCC = Squamous Cell Carcinoma.

		MANOVA			Mahalanobis Distances		
		BCC	BEN	IEC	BCC	BEN	IEC
BEN	*p*-Value	0.001			9.7 × 10^−47^		
	FPR	1.8%			2.8 × 10^−42^%		
IEC	*p*-Value	0.756	0.004		0.228	1.6 × 10^−27^	
	FPR	-	5.7%		37.9%	2.7 × 10^−23^%	
SCC	*p*-Value	0.030	0.001	0.023	0.738	2.9 × 10^−22^	0.292
	FPR	22.2%	1.8%	19.1%	-	2.9 × 10^−10^%	49.4%

**Table 2:** Descriptive statistics for the asymmetry indices of each of the samples. For space restrictions, FPR values were excluded from the present table considering all *p*-values were far below the 3σ threshold. L (0.05) = Lower bound 95% confidence interval; U (0.95) Upper bound 95% confidence interval; √BWMV = Square Root of the Biweight Midvariance.

	Shapiro							
	*w*	*p*	Min.	L (0.05)	Median	√BWMV	U (0.95)	Max.
BCC *	0.782	2.2 × 10^−^^16^	0.131	0.179	0.292	0.099	0.554	1.064
IEC *	0.647	2.6 × 10^−^^12^	0.168	0.186	0.292	0.081	0.621	1.285
SCC *	0.566	1.2 × 10^−^^14^	0.097	0.172	0.270	0.084	0.572	1.390
BEN	0.616	2.2 × 10^−^^16^	0.134	0.183	0.239	0.046	0.373	1.213
Cancer *	0.679	2.2 × 10^−^^16^	0.097	0.183	0.289	0.093	0.554	1.390
Benign	0.616	2.2 × 10^−^^16^	0.134	0.183	0.239	0.046	0.373	1.213

* Malignant (cancerous) samples.

**Table 3:** Multivariate Analysis of Variance (MANOVA) and Mahalanobis distance testing to assess the degree of statistical differences between sample morphologies combining shape information and asymmetry.

		MANOVA			Mahalanobis Distances		
		BCC	BEN	IEC	BCC	BEN	IEC
BEN	*p*-Value	0.001			3.6× 10^−45^		
	FPR	1.8%			1.0 × 10^−^^40^%		
IEC	*p*-Value	0.814	0.001		0.242	3.4 × 10^−^^27^	
	FPR	-	1.8%		48.3%	5.6 × 10^−^^23^%	
SCC	*p*-Value	0.058	0.001	0.021	0.452	4.0 × 10^−^^23^	0.051
	FPR	31.0%	1.8%	18.1%	-	5.6 × 10^−^^19^%	29.2%

**Table 4:** Overall evaluation metrics on test sets using Support Vector Machines for the classification of Benign and Malignant lesions. AUC = Area Under the precision–recall Curve. The combined EFA & Asymmetry category represents PCA dimensionality reduction techniques performed on both EFA coefficients and Asymmetry indices, prior to SVM training.

Training Variables	Accuracy	Precision	Recall	F-Statistic	AUC
Asymmetry	0.690	0.646	0.447	0.528	0.696
EFA Coefficients	0.772	0.887	0.717	0.794	0.693
EFA & Asymmetry	0.765	0.883	0.711	0.788	0.685
Combined EFA & Asymmetry	0.786	0.915	0.717	0.804	0.735

**Table 5:** Confusion Matrix calculated on test sets using the Combined EFA & Asymmetry dataset.

		True	
		Benign	Malignant
**Predicted**	**Benign**	71.67%	10.53%
	**Malignant**	28.33%	89.47%

## Data Availability

All data used in the present study originate from the Dermofit dataset and are available for purchase through the University of Edinburgh; https://licensing.edinburgh-innovations.ed.ac.uk/i/software/dermofit-image-library.html (accessed on 27 April 2021).

## Supplementary Materials

The following supporting information can be downloaded at: https://www.mdpi.com/article/10.3390/jcm11154392/s1, Supplementary File: R code for the calculation of Elliptic Fourier Coefficients.
